# Regional data exchange to improve care for veterans after non-VA hospitalization: a randomized controlled trial

**DOI:** 10.1186/s12911-019-0849-1

**Published:** 2019-07-04

**Authors:** Brian E. Dixon, Ashley L. Schwartzkopf, Vivian M. Guerrero, Justine May, Nicholas S. Koufacos, Andrew M. Bean, Joan D. Penrod, Cathy C. Schubert, Kenneth S. Boockvar

**Affiliations:** 1 Department of Veterans Affairs, Health Services Research & Development Service, Center for Health Information and Communication, 1481 W. 10th St, 11H, Indianapolis, IN 46202 USA; 2 Indiana University, Fairbanks School of Public Health, 1050 Wishard Blvd, Indianapolis, IN 46202 USA; 3 Regenstrief Institute, Center for Biomedical Informatics, 1101 W 10th St, Indianapolis, IN 46202 USA; 4 Department of Veterans Affairs, James J. Peters VA Medical Center, 130 W Kingsbridge Rd, Bronx, NY 10468 USA; 5 Icahn School of Medicine at Mount Sinai, 1 Gustave L. Levy Pl, New York, NY 10029 USA; 6 Indiana University, School of Medicine, 1101 W. 10th St, Indianapolis, IN 46202 USA

**Keywords:** Health information exchange, Veterans health, Reminder systems, Community networks, Hospitalization, Emergency service, Hospital

## Abstract

**Background:**

Coordination of care, especially after a patient experiences an acute care event, is a challenge for many health systems. Event notification is a form of health information exchange (HIE) which has the potential to support care coordination by alerting primary care providers when a patient experiences an acute care event. While promising, there exists little evidence on the impact of event notification in support of reengagement into primary care. The objectives of this study are to 1) examine the effectiveness of event notification on health outcomes for older adults who experience acute care events, and 2) compare approaches to how providers respond to event notifications.

**Methods:**

In a cluster randomized trial conducted across two medical centers within the U.S. Veterans Health Administration (VHA) system, we plan to enroll older patients (≥ 65 years of age) who utilize both VHA and non-VHA providers. Patients will be enrolled into one of three arms: 1) usual care; 2) event notifications only; or 3) event notifications plus a care transitions intervention. In the event notification arms, following a non-VHA acute care encounter, an HIE-based intervention will send an event notification to VHA providers. Patients in the event notification plus care transitions arm will also receive 30 days of care transition support from a social worker. The primary outcome measure is 90-day readmission rate. Secondary outcomes will be high risk medication discrepancies as well as care transitions processes within the VHA health system. Qualitative assessments of the intervention will inform VHA system-wide implementation.

**Discussion:**

While HIE has been evaluated in other contexts, little evidence exists on HIE-enabled event notification interventions. Furthermore, this trial offers the opportunity to examine the use of event notifications that trigger a care transitions intervention to further support coordination of care.

**Trial registration:**

ClinicalTrials.gov NCT02689076. “Regional Data Exchange to Improve Care for Veterans After Non-VA Hospitalization.” Registered 23 February 2016.

## Background

### Background and rationale

The U.S. healthcare system is characterized by high sub-specialization relative to international comparisons [1], creating significant need for coordination among the various types and levels of provider services. Patients receive care from a variety of providers, with some providers being outside of any one, organized network [2]. Moreover, individuals increasingly manage their health using a variety of interventions at home as well as the Internet [3]. Delivering high quality, coordinated care therefore requires that providers access, manage, and share information efficiently.

Managing a patient’s health information for care coordination activities, however, is challenging. In a study of internal medical residents, researchers observed that providers spent between 5 and 9% of their time looking for information on a patient [4]. In a survey of U.S. patients, 27% of respondents reported that during a medical visit their test results were either not available, or that duplicate tests were ordered; and 17% of patients reported that information was not shared among their multiple care providers [5]. Respondents from Australia, Canada, France, and other nations indicated similar information gaps with respect to care coordination [5].

To optimize care coordination, providers require health information technologies that facilitate access to information on their patients, regardless of its source. Health information exchange (HIE) is defined as the electronic transfer of clinical, administrative, or other information necessary for the delivery of health care across diverse systems or organizations [6]. Most often HIE takes the form of an information system transaction mediated by technical standards, such as the electronic reporting (or pushing) of a result from a laboratory information management system to an electronic health record (EHR) system using the Health Level Seven (HL7) messaging standard. Other times HIE involves querying (or pulling) information from another system, such as retrieval of the patient’s last radiological study performed by another provider using the Digital Imaging and Communications in Medicine (DICOM) standard.

Event notification is a form of HIE involving the electronic reporting (or pushing) of information pertaining to a clinical event from one provider to another facilitated by a messaging standard. Notification usually pertains to acute care events (e.g., hospitalization, emergency care), and the notifications are typically sent to primary care providers responsible for coordination of care [7]. To date, there has been one quantitative study of event notification sent to ambulatory providers for acute care events over a 3.5 year period using a regional HIE network [8]. The study found a statistically significant 2.9% reduction in 30-day readmissions observed during the intervention period. While promising, the evidence represents a single before-after cohort study. More research is necessary to establish robust evidence on the benefits of event notification services as a form of HIE for care coordination.

### Objectives

The objectives of this study are to 1) examine the effectiveness of event notification on health outcomes for older adults who experience acute care events, and 2) compare approaches to responding to event notification.

### Trial design

We will implement a multi-year, mixed methods study involving a clinical trial of the HIE intervention and qualitative examination of its implementation. A 2-site cluster-randomized trial will examine the impact of event notification introduced into multiple primary care medical homes part of a large integrated health system.

**Fig. 1:**
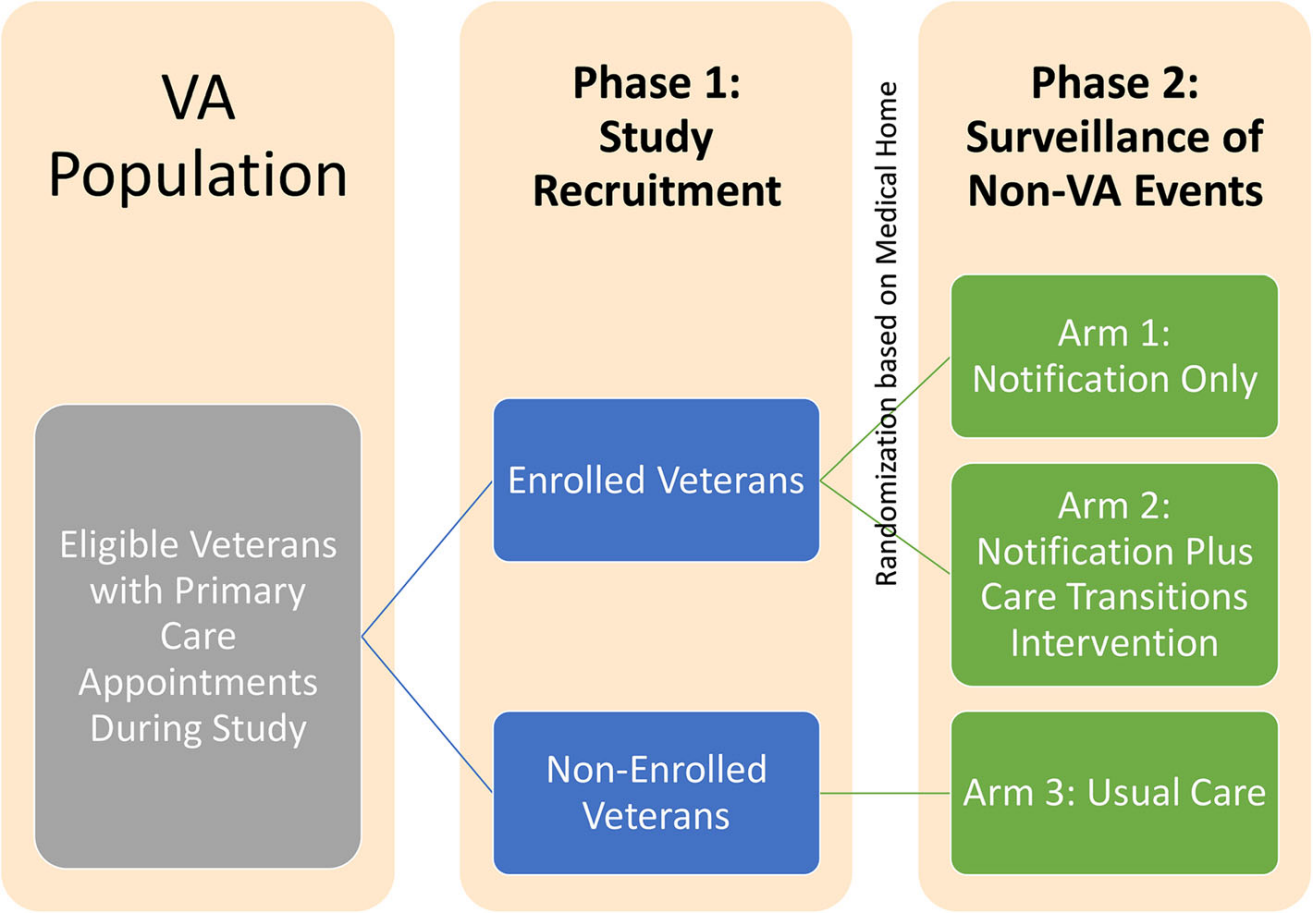
Overview of the study population, study arms, and phases of the study. VA = Veterans Affairs

The trial involves three arms recruited in two phases as depicted in Fig. 1. In Phase 1, we will identify and enroll active subjects into one of two groups: 1) event notification only, in which the primary care provider is alerted when the subject has an acute care event outside the integrated health system; or 2) event notification plus geriatric care transitions intervention, in which the primary care provider is alerted in parallel with a geriatric care coordinator who will facilitate a standardized care transitions intervention for 30 days following discharge from the acute care facility. Assignment is based on the patient’s primary care medical home, which will be randomized to one of the arms. In Phase 2, we will retrospectively identify and capture data on a third group of concurrent control patients for which no notification or post-acute care transitions intervention was utilized. Comparisons will be performed between each intervention group and the control group as well as between the two intervention groups.

In addition to the quantitative trial, we will further perform a qualitative assessment examining the implementation of event notification as well as the care transitions intervention. This component of the study will examine the facilitators and barriers of the HIE-based intervention as well as care coordination following the transition of care. Interviews with providers and patients will elicit details about socio-technical dimensions that cannot be measured quantitatively using data from the trial.

## Methods

### Study setting

The study takes place in the context of the Veterans Health Administration (VHA). The VHA is the largest integrated health care system in the United States, providing care at 1,243 health care facilities, including more than 170 medical centers and 1,000 outpatient clinics, serving 9 million enrolled veterans each year.

The VHA has a need to improve care coordination between VHA-based providers and non-VHA providers who practice in community hospitals, private clinics, and other networks such as federally qualified health centers. In a survey of non-VHA providers part of a practice-based research network [9], respondents reported poor communication with VHA colleagues, and their interactions were perceived to be with a “system” rather than a colleague. Moreover, VHA is under pressure to provide more non-VHA care to Veterans in an effort to improve access to care following a series of appointment scheduling challenges [10].

Historically the VHA spent around 10% of its total health care dollars on care delivered to veterans outside of the VHA’s network of providers [10]. Recent legislation-- the CHOICE Act of 2014 [11] and MISSION Act of 2018 [12] -- directs the VHA to expand out-of-network care to veterans. Since the passage of the CHOICE Act, the VHA has authorized nearly 6 million non-VHA visits for nearly 2.4 million unique Veterans, nearly one-third its total population. As non-VHA care visits increase, VHA needs to strengthen care coordination processes to control quality as well as costs.

Two medical centers within the VHA are included in the study. The first medical center is the James J. Peters VA Medical Center (JJP VAMC) located in the Bronx, New York. The JJP VAMC cares for more than 26,000 patients annually via a tertiary care facility providing comprehensive inpatient as well as outpatient care services in addition to four community-based outpatient clinics. The second medical center is the Richard L. Roudebush VA Medical Center (RLR VAMC) located in Indianapolis, Indiana. The RLR VAMC serves more than 62,000 patients annually and consists of a tertiary care facility providing comprehensive inpatient as well as outpatient care services in addition to three community-based outpatient clinics. Both medical centers also serve as teaching hospitals and regional referral sites.

The study utilizes cluster randomization in which the interventions will be randomly allocated to the primary care teams distributed across the two sites. Roughly half of the primary care teams at each site will be randomly assigned to the event notification-only arm. The other half will be allocated to the event notification plus geriatric care transitions intervention. Clustering within primary care teams prevents bias with respect to providers who might, once they become aware that some patients are receiving a care transitions intervention, try to emulate that service for other patients who may be allocated to a notification-only arm.

### Eligibility criteria

A veteran is eligible for this trial if he or she 1) receives primary care at the Bronx VA Medical Center or Indianapolis VA Medical Center; 2) is 65 years or older; 3) agrees to consent to HIE between the VHA and non-VHA providers; and 4) has utilized any non-VHA services (including nursing, lab, physician, pharmacy, and/or hospital services) within the previous two years according to records in external HIE network or self-report.

A veteran is excluded if he or she receive services from the Geriatric Resources for Assessment and Care of Elders (GRACE) Team Care [13, 14], a care management model previously implemented within the geriatric service at the Indianapolis VA Medical Center [15], since this program is very similar to the study’s care transitions intervention. If a veteran becomes enrolled in GRACE after enrollment in this trial, he or she will be withdrawn from this trial and notified by mail of the conflict.

### Interventions

**Fig. 2:**
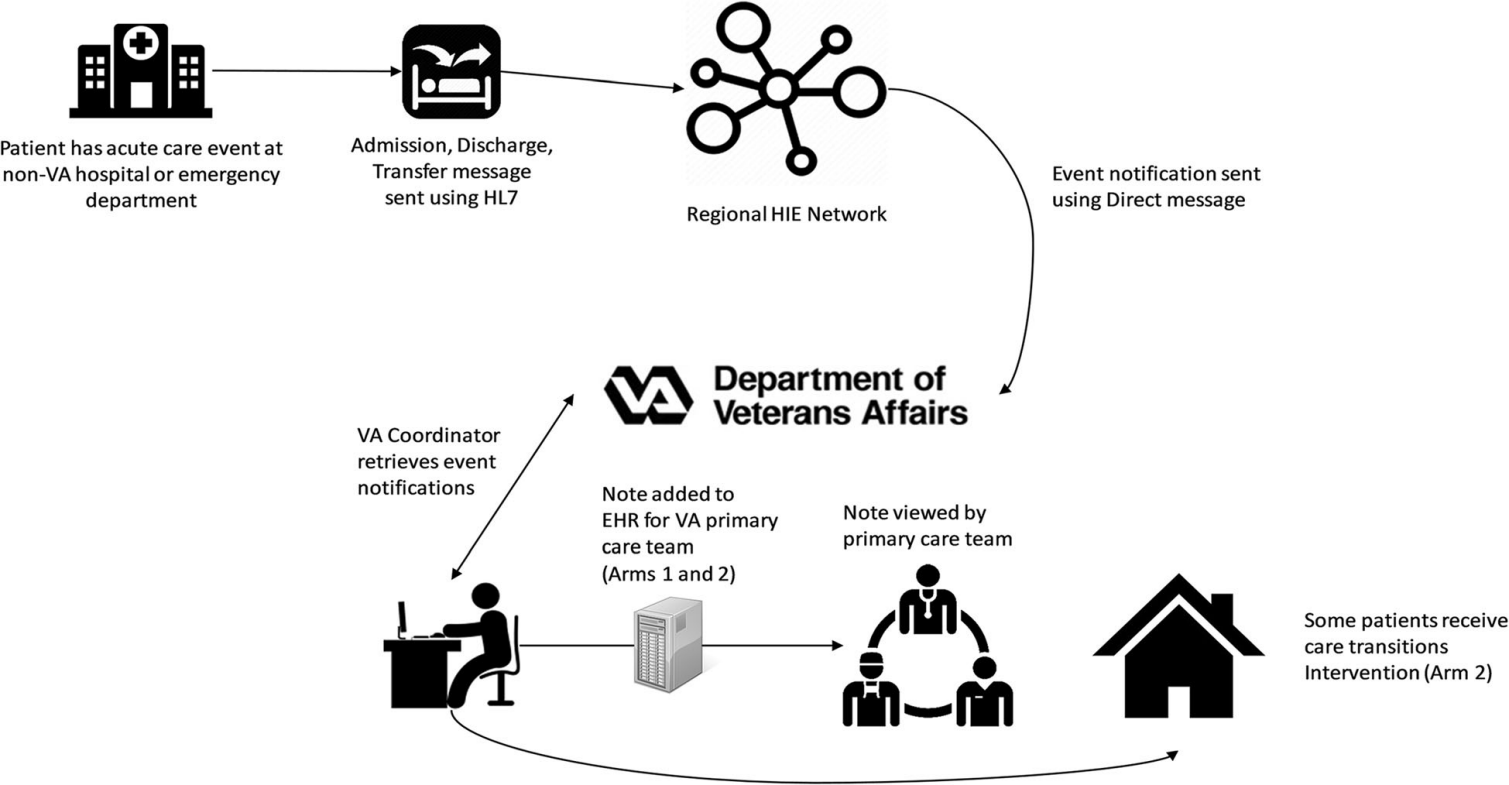
Information flow when a patient has a non-VA acute care encounter. Event notifications are sent via the regional HIE network to the VA for patients in all arms. A coordinator at the VA enters the notification as a note into the EHR for the primary care team to review and co-sign for patients enrolled in arms 1 and 2. For patients in arm 2, a care transition intervention is activated for 30 days post-discharge. VA = Veterans Administration; HIE = Health information exchange; HL7 = Health Level 7; EHR = Electronic health record

This study involves two distinct interventions: 1) an HIE-based event notification message transmitted to the patient’s primary care team when a veteran has a non-VHA acute care encounter; and 2) a care transitions intervention, in which a geriatric care coordinator facilitates follow-up care with a veteran for 30 days following discharge from the acute, non-VHA care facility. These interventions are depicted in Fig. 2.

The first intervention, event notification, is provided to patients in study arms 1 and 2. Upon utilization of non-VHA acute care, such as an inpatient admission or emergency department (ED) visit, an HL7 admission-discharge-transfer (ADT) message is electronically sent from the acute care facility to an HIE network connected to the two participating VHA sites. In New York, this is the Bronx RHIO. In Indianapolis, this is the Indiana Health Information Exchange. Descriptions of these networks is provided at the end of this section. Each HIE network responds to the ADT message by notifying (or alerting) the VHA that an enrolled patient has visited a non-VHA care facility.

**Fig. 3:**
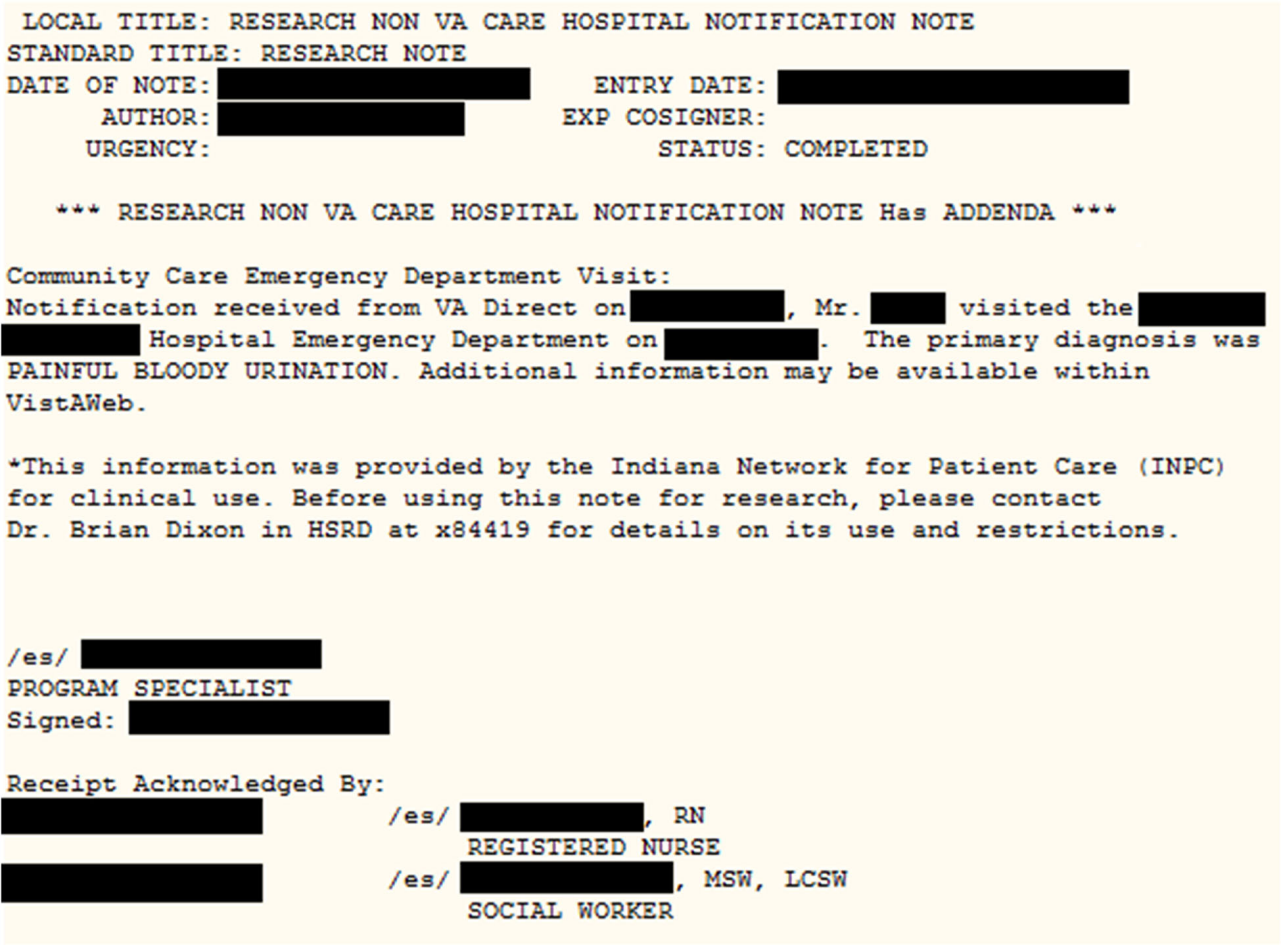
Screenshot of a non-VA encounter note entered into the VA EHR for a patient in either study arm 1 or arm 2. The note must be acknowledged by a member of the patient’s primary care team

The ADT alert triggers the event notification intervention. Following notification of the non-VHA acute care event, study coordinators at the two VHA sites send an internal electronic note within the VHA’s EHR system to the veteran’s primary care medical home team (referred to within the VHA as a PACT team). This note, which becomes part of the veteran’s medical record, identifies the non-VHA care facility and provides information on the reason for the visit or chief complaint. It further provides details on the external provider and how to contact them. An example message is depicted in Fig. 3.

The second intervention, a standardized care transitions intervention facilitated by a geriatric care coordinator, is provided to study patients in arm 2 only. In addition to alerting the PACT team, study coordinators at the two sites provide an electronic notification of the non-VHA acute care event to a geriatric care coordinator. While the background of the care coordinator can vary, both sites used geriatric social workers to fill this role. The care coordinators review the veteran’s medical record in the EHR, and they contact the veteran to schedule an in-home visit within 1–5 days following discharge from the external hospital or ED. If a patient is discharged to a rehabilitation setting, the home visit should occur within 1–5 days after rehabilitation facility discharge.

After the home visit, care coordinators perform at least 3 follow-up phone calls for ongoing education and counseling over the subsequent 30 days. The home visit and any subsequent phone calls are documented in clinical notes within the VA EHR system.

The care transitions intervention follows a standardized model developed by Coleman [16, 17] and focuses on activation of the patient to manage his or her own care. The model consists of four pillars for transition care, including 1) understanding and self-management of VHA and non-VHA medications; 2) diagnosis-specific education and counseling, and understanding “red flag” symptoms that require medical attention; 3) creation of a patient-centered record containing contact information, conditions, medications, and advance directives; and 4) self-management of follow-up care within the VHA and non-VHA systems. Care coordinators consider themselves coaches to facilitate veteran’s active, independent self-management of health rather than passive recipients of service. They prefer that veterans schedule follow-up appointments, resolve medication problems, initiate behavioral changes, and complete rehabilitation exercises themselves. Yet the coordinators emphasize they are available for help and encouragement during the 30-day period.

The Bronx Regional Health Information Organization (Bronx RHIO) is a HIE that includes hospitals, physician offices, and long-term care organizations, among others. These providers collectively deliver a majority of the care received by the 1.4 million residents of the Bronx, including over 95% of the borough’s annual hospital discharges, more than 600,000 annual emergency care visits, and 4.5 million annual ambulatory care visits. The Bronx RHIO facilitates secure, interoperable HIE within the borough and connects its jurisdiction to the rest of the state via the Statewide Health Information Network for New York. The providers at the Bronx VA Medical Center have had access to the Bronx RHIO since 2008.

The Indiana Health Information Exchange (IHIE) is a non-profit organization that facilitates health data exchange, including governance as well as security, across an information technology network that connects over 140 hospitals, long-term care facilities, rehabilitation centers, community health clinics and other healthcare providers in Indiana. This network serves more than ten million patients and over 30,000 physicians throughout the country. On top of the network, IHIE offers a variety of patient and population information services such as clinical messaging, analytics, and ADT alerts. IHIE has exchanged data with the VHA since 2011 when it began sending summary of care documents for veterans enrolled in the Veterans Lifetime Electronic Record program [18].

### Outcomes

The primary outcome measure is VHA and non-VHA hospital admission or readmission 90 days after non-VHA hospital or ED discharge (or, if the patient is not discharged home, 90 days after discharge home from a rehabilitation facility). Data on VHA and non-VHA hospital use will be retrieved through the regional HIE partner, the EHR system, and the corporate VHA data warehouse. We chose hospital admission or readmission as the primary outcome because this measure is 1) the most commonly reported outcome in studies of geriatric care transitions interventions [16, 19–22] and a common outcome reported in studies of HIE [23, 24], enabling comparison of study findings with others; 2) frequent; 3) important to patients, providers, and policymakers; and 4) can be ascertained objectively with low risk of bias.

Secondary outcome measures were selected to provide information on intermediate outcomes addressed by the HIE and care transitions intervention components. They include:

- Post-acute care follow up. Timely VHA follow-up will be defined as a follow-up visit with any VHA provider within 30 days of non-VHA hospital discharge or ED visit. Timely phone call will be defined as VHA PACT phone call within 7 days of non-VHA hospital discharge or ED visit. We will obtain VHA encounter and phone call dates from the EHR system, and non-VHA encounter dates will be obtained from the HIE networks.
- High-risk medication discrepancies, defined as the number of discrepancies in medications classified as high risk for hospitalized older adults, including opioid analgesics, insulin, non-steroidal anti-inflammatory drugs, digoxin, antipsychotics, sedatives/hypnotics, and anticoagulants [25–27]. We will obtain a count of high-risk discrepancies based on medical record review and patient or caregiver interview 30 days after non-VA hospital discharge.
- Care Transitions Measure. This measure of condition self-knowledge and transitional care quality from the patient’s perspective is ascertained by patient or caregiver interview 30 days after non-VHA hospital discharge. A 3-item questionnaire, previously developed and validated [28], will be used. The questionnaire includes items such as “After I left the hospital, I had all the information I needed to be able to take care of myself” with the response options strongly agree, agree, disagree, strongly disagree, and don’t know.
- Process measures, which provide information on processes influenced by HIE-notifications and by care transitions intervention components. We will calculate frequency of HIE-access by providers (login percent per encounter; login duration; tabs browsed) through system audits provided by Bronx RHIO and Indiana HIE. We will calculate frequency of veteran contacts (in person and telephone) by PACT team and care transitions coordinator, using EHR chart review and coordinator logs. We will calculate the percentage of care transitions intervention components delivered that were indicated using coordinator logs.

Qualitative outcomes will include the following:

- Perceptions of providers as well as veterans on the event notification alerts sent following an acute care event. Do patients perceive these alerts as valuable? Do providers view the alerts as helpful or a hindrance to the care they provide? How can the alerts be delivered in a manner that better supports workflow and patient reengagement following an acute care event?
- Why do veterans seek acute care outside of the VHA system? While the VHA has enabled greater access to VHA providers, it is not always clear why veterans choose to receive non-VHA services during an acute care event.

### Participant timeline

**Fig. 4:**
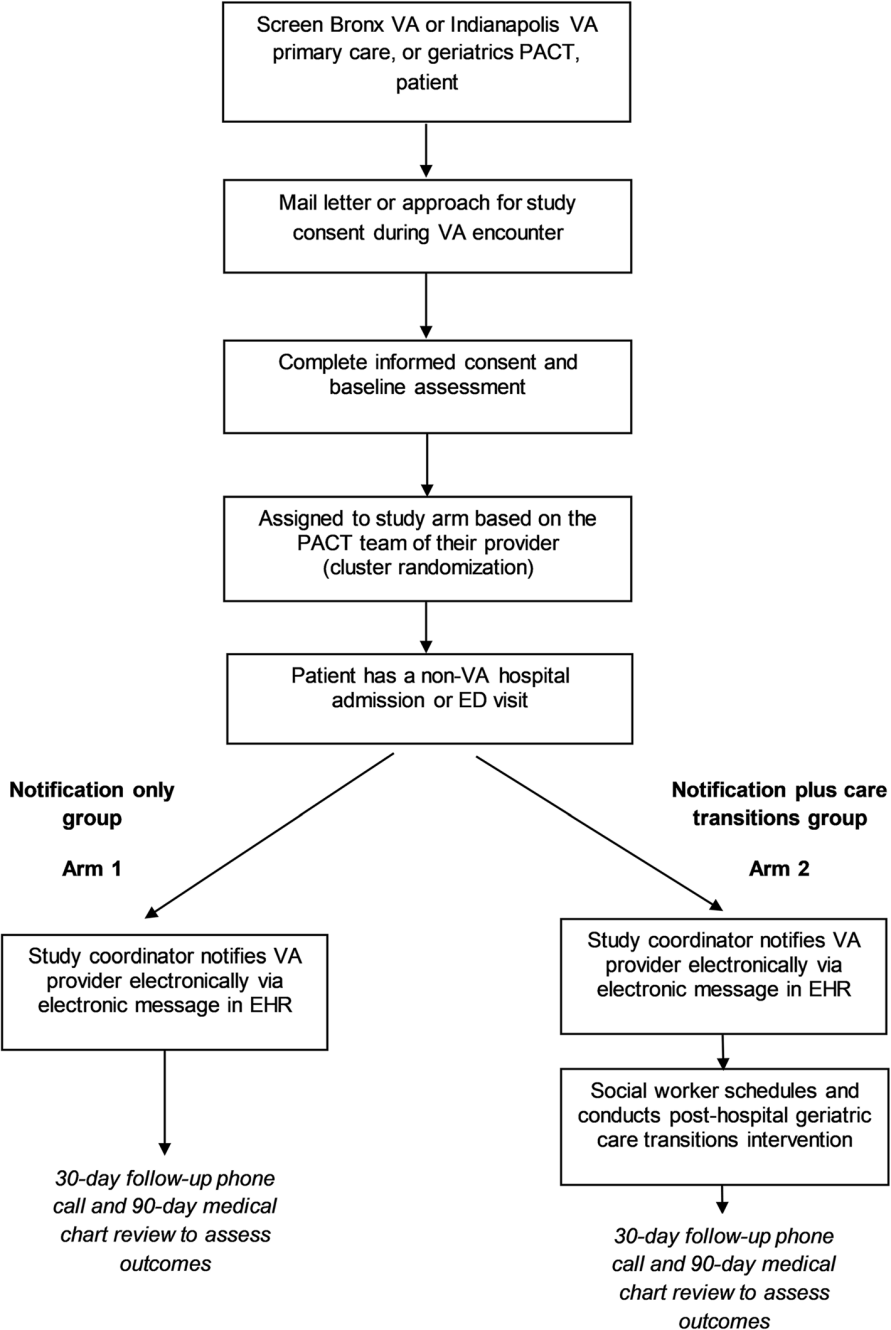
Overview of patient screening, enrollment, and activation procedures performed by research staff. The figure distinguishes between study arm 1 (notification only) and arm 2 (notification plus care transitions intervention)

The enrollment and participant timeline is depicted in Fig. 4. Veterans who actively enroll in the study during Phase 1 are placed into one of two arms based on their primary care provider’s PACT team per the cluster design. Non-VHA acute care visits are monitored for both arms using a public health surveillance approach. If a non-VHA visit is detected, the response is driven by the arm into which his or her PACT team was randomized. Regardless of arm, a research team member will contact the veteran 30 days after discharge from the non-VHA facility to conduct an interview over the phone. In the case where a patient is discharged to a rehabilitation or nursing facility, follow-up will occur 30 days after discharge from that facility. A medical chart review is conducted for the 90-day period following discharge to ascertain readmission to a hospital or emergency room visit as well as other secondary outcomes.

### Sample size

Our sample size estimate is based on the primary outcome. Reports of randomized studies of geriatric care transitions interventions have reported 25–45% reductions in hospital readmissions [17, 20, 29]. Based on prior work as well as pilot data gathered within the VHA (unpublished), we postulate that the notification-only group as well as the usual care group will have a 40% readmission rate while the notification-plus-coordination group will have a 26% readmission rate in the 90-day post-discharge period (i.e., a clinically significant 35% reduction).

There are several features of the study that support this large potential effect. First, the targeted patients are at high risk of readmission because they are older veterans who utilize both VHA and non-VHA services, who have been shown in previous studies to have significantly higher readmission rates after acute illness. Second, in contrast to veterans hospitalized in the VHA who routinely receive PACT post-discharge telephone calls and follow-up, this group is naïve to any coordination intervention because PACT teams normally do not respond to non-VHA hospital encounters because they do not know about them. Thus, we postulate more than an incremental effect.

To account for possible dependence of the outcome among patients cared for by the same PACTs (clustering), we estimated the design effect [30] based on an average number of subjects per PACT = 7. Assuming the intraclass correlation is 0.05 (based on pilot data and prior literature [31]), we need to observe a total of 466 patients with non-VHA hospitalizations or ED visits (155 in the notification-plus-coordination group and 311 combined in the notification-only and usual care groups) to detect an effect size of 0.35 with 80% power and a two-tailed test at 5% significance. If the intraclass correlation is smaller (e.g., 0.04), which may be the case for a group of patients whose care is fragmented and perhaps less influenced by primary care team clustering, then the power will be higher.

In order to achieve the target sample size, we will enroll 300 patients at each site, with an estimated 25–30% of enrolled veterans experiencing non-VHA hospital encounters. We anticipate 10–15% attrition due to death or loss to follow up given the average age of veterans in the target population.

### Recruitment

We will employ a number of strategies to maximize recruitment. The VHA Office of Research & Development requires that Veterans first be contacted in writing before being approached by the study team. Using a semi-automated method in which we identify potentially eligible veterans using EHR data in combination with the scheduling system similar to prior VHA studies [32], we will send letters to veterans with upcoming VA primary care appointments whose medical chart pass initial screening. Next we will contact veterans who receive a letter and schedule in person appointments to complete screening and enrollment. We will further contact PACT teams and providers directly. Interested Veterans who inquire during a clinic visit about participation will be able to contact the study team for eligibility screening during business hours and answered by voicemail at all other times.

### Allocation

We will cluster randomize by PACT team to prevent a team from having patients in both treatment groups and reduce contamination. Prior to initiating enrollment, the lead investigator (KSB) will assign PACTs to notification-plus-coordination or notification-only groups, using lists of computer-generated random numbers in a 1:1 ratio, with separate lists for the Bronx VA and Indianapolis VA, in order to maintain balance in groups within each study site. There are 25 primary care PACTs in the Bronx and 41 in Indianapolis, and each PACT will keep its group assignment for the duration of the study. In Phase 1, enrolled patients will be assigned to notification-plus-coordination or notification-only groups according to their assigned PACT team.

### Data collection methods

We will collect data on veterans using a variety of methods. At baseline as well as 30 days following an acute care event, we will capture data from the Veteran or a caregiver using a questionnaire administered by a trained Research Assistant. The questions asked will pertain to a veteran’s demographics, high risk medications, functional status, and mental health status. High risk medication use will be assessed using a series of questions asking whether the veteran takes blood thinners, medicine for diabetes, pain medications, sleep medications, or mental health medications. Functional status will be assessed using the Katz Activities of Daily Living (ADL) [33] as well as the Lawton Instrumental ADL [34] screeners. Mental health status will be assessed using the Short Portable Mental Status Questionnaire (SPMSQ) [29].

At 30 days following an acute care event, we will also ask questions about the acute care event, care transitions following the acute care event, and perceptions of HIE. veterans will be asked to recall the reason for their acute care event as well as the reason why they chose to use a non-VHA hospital. Care transitions will be assessed using a validated measure of condition self-knowledge and transitional care quality from the patient’s perspective [28]. Perceptions of HIE will ascertained using questions that ask veterans how they feel about their health information being shared electronically between their VHA and non-VHA providers. We will also ask whether they feel that sharing information electronically had any effect on the quality of care they received.

At 90 days following a non-VHA acute care event, trained RAs will extract data from the veteran’s electronic medical records to ascertain primary outcomes, including readmissions following the acute care event as well as health status. The medical records will be obtained from the VHA central data warehouse as well as the Bronx RHIO and Indiana HIE. This will ensure broad capture of data from all available sources pertaining to the non-VHA event as well as follow up within the VHA health system. The RAs will use a standardized template for electronic chart review. Before beginning record reviews and periodically during the study, the research staff will code 5–10 previously coded medical records. We will retrain if accuracy per chart is less than 95%.

During the intervention period, we will interview PACT team members about their experiences with the event notifications they receive in the EHR. We will interview doctors, nurses, medical assistants, and other clinical staff regarding whether they have noticed the alerts and what steps they take following an alert. We will further ask team members about their perceptions of the alerts, such as whether they are barriers or facilitators in care coordination processes. Interviews will further provide an opportunity to capture feedback on the notifications as well as the processes in the VHA that could enable or prevent widespread implementation of event notifications across the VHA health system. These interviews will be conducted using a semi-structured interview guide to be developed by the investigators and influenced strongly by the Consolidated Framework for Implementation Research (CFIR), which is the preferred framework used within the VHA health system [35].

### Data management

All study data will be captured and stored within the VHA using standard tools available to VHA researchers. Information captured from veterans or extracted from medical records will be stored in a secure study database hosted on a password-protected server accessible only to authorized study personnel. The randomization status for each cluster will be kept in a secure location accessible only to study investigators but not the RAs and project managers to ensure blinding.

The database will contain separate tables that will store individuals’ data pertaining to eligibility, enrollment, activation (e.g., non-VHA acute care events), baseline information, 30-day follow up information, 90-day follow up information, and study completion or withdrawal. The database will enable tracking of individuals who are believed to be eligible and whom the study team mailed information about participation, as well as those who met with an RA but were found to be ineligible for study enrollment. This database will be used to drive trial processes as well as store data for planned analyses, both primary and secondary. Periodic review of data completeness and instruments used to capture data will be performed by the study team to ensure quality of data collection and analysis. Furthermore, prompts will be developed in data entry forms for out-of-range and inconsistent entries, to prompt immediate correction.

Informed consent will be obtained from all participants and confidentiality will be ensured. The database as well as hard copies of patients’ informed consent documents and other study files will be secured and stored according to the regulations of the VHA Research & Development Office, institutional ethics board, as well as the Health Insurance Portability and Accountability Act (HIPAA). Study records will be maintained and expunged in accordance with the VHA Records Control Schedule.

Open-ended interview data will be transcribed and coded by VHA research staff. Codebooks will be developed collaboratively by the investigators, and coding quality will be routinely assessed by the study team to ensure consistency across transcripts. Regular meetings to review transcript snippets and coding progress will ensure harmonization of approaches across coders, and disputes will be resolved by the principal investigator.

### Statistical methods

All analyses will be performed as intention-to-treat (ITT), i.e. all outcomes will be included on patients assigned to treatment groups, even if they deviate from the group’s intervention. We will use a generalized linear mixed (GLM) model to test the effect on the primary outcome of 90-day hospital admission or readmission (e.g., 0 = no, 1 = yes). The logit link of the binary outcome for each observation will be modeled as a linear model, with the PACT team used as a random effect and group assignment (e.g., 0 = notification only or no notification, 1 = notification-plus-coordination) as a fixed effect. Inclusion of random effects in the model accounts for possible within-cluster correlation of the outcome since patients will be clustered by PACT.

To estimate the intervention effect after accounting for patient characteristics, especially those found to be imbalanced between study arms from the preliminary analyses, other covariates will be added as fixed effects to the model. These covariates may include: age, gender, non-VHA hospital, number of pre-admission medications, chronic illness count, discharge diagnosis, and length of stay for the index admission (with ED visits coded as 0 days). In addition to bias reduction, this analysis may increase the power for detecting the intervention effect by reducing the unexplained variance of the outcome and add insight into factors that predispose these patients to the outcome.

A similar approach will be used to test the intervention effect on secondary outcomes. For each secondary outcome, an appropriate distribution or link function will be chosen for the GLM model, e.g. logit link for the binary outcome of scheduled follow-up, Poisson or Negative Binomial distribution for the counts of high-risk medication discrepancies, identity or other links for Care Transitions Measure depending on the distribution of scores.

All analyses will be carried out using the most recent version of SAS (Carey, North Carolina, USA) available to VHA researchers using the VHA computing environment. For all tests, we will use 2-sided *p*-values with alpha = < 0.05 level of significance. Qualitative coding and analysis will be conducted using the most recent version of NVivo (QSR International; Doncaster, Victoria, Australia).

### Ethics

This protocol, along with all informed consent documents, questionnaires and data collection templates, will be reviewed and approved by the Institutional Review Board (IRB) of Indiana University as well as the VA Research & Development Committee at both the Indianapolis VA Medical Center and the Bronx VA Medical Center.

Subsequent to initial review and approval, the responsible IRBs and VHA research committees will review the protocol at least annually. The investigators will make safety and progress reports to the IRBs at least annually and within 6 months of study termination or completion. These reports will include the total number of participants enrolled, serious adverse events, reportable events (e.g., death of a patient unrelated to the study), and summaries of each DSMB [Data Safety and Monitoring Board] review of safety and/or efficacy.

Any modifications to the protocol which may impact on the conduct of the study, potential benefit of the patient or may affect patient safety, including changes of study objectives, study design, patient population, sample sizes, study procedures, or significant administrative aspects will require a formal amendment to the protocol. Such amendment will be agreed upon by the VHA Health Services and Research Department (HSR&D, the funder) and approved by the IRBs and VHA research committees prior to implementation in accordance with VHA regulations.

### Dissemination

Primary and secondary outcomes will be analyzed and published via peer-reviewed publications. Specific publications will be determined and written by the investigators as preliminary as well as final data become available for analysis. Once accepted, publications will be reported to the VHA HSR&D Service and made available publicly via PubMed Central per VHA regulations.

In addition to peer-reviewed manuscripts, the study team further intends to present preliminary and initial analyses at scientific conferences such as meetings organized by the American Medical Informatics Association, the American Geriatrics Society, and the Agency for Health Research and Quality. Furthermore, the study team will distribute findings as well as lessons learned to VHA operations teams to enable adoption and implementation of high quality care transitions throughout the VHA system. Intended stakeholders include the VHA Office of Patient Care Services- Geriatrics and Extended Care Services, Case Management and Social Work, Office of Connected Care, and the VHA Office of Health Information.

## Discussion

Our study is innovative in its use of a trial design to examine the impact of event notifications, enabled by HIE, on health outcomes. HIE has been associated with a number of benefits [36], including a reduction in expensive imaging studies [37], improved population level immunization rates [38] and completeness of disease reporting to public health authorities [39]. Given the ability of HIE to facilitate access to patient information, especially after a handoff or transfer of care, there exists a strong theoretical case for HIE to impact care coordination activities [40], such as reintegration into primary care following an acute care episode. However, recent systematic reviews of HIE [36, 41] do not include such studies. This may be due to the fact that the only prior study examining event notification [8] utilized a cohort design found to be insufficient for inclusion in a systematic review. Our study is designed to generate high quality evidence on event notification, evidence needed for assessing the impact of HIE services on patient and population outcomes in future reviews.

Our study is further innovative in its combination of arms that enable examination of a care transitions intervention pioneered by Coleman et al. [16] beyond event notification enabled by HIE. Prior studies in multiple countries have examined interventions designed to improve care transitions for older adults [42–46], yet most focus on steps that can be taken by clinical staff within emergency department or hospitals prior to discharge. Few interventions can be implemented to initiate care transitions following discharge, because there are many breakdowns in communication with external primary care providers once the patient leaves a facility. The growth in HIE networks provides an opportunity to identify a discharge event and initiate timely follow up by primary care providers. This study is designed for sufficient power to examine not only the benefit of event notification but also the additional benefit of a care transitions intervention that can activate patients to better self-manage their complex disease and medication regimens at home.

Although this study is conducted within the VHA system, the findings should be applicable to other health systems within the U.S. as well as global health systems. There is nothing inherent in the study design that precludes implementation in other settings. With respect to the informatics systems, ADT systems are widespread in health care systems as they drive many EHR and decision support system functions. Furthermore, patients cross “system” boundaries in every country, including care seeking at public and private hospitals as well as “in-network” and “out-of-network” facilities. Prior studies examining global health systems find a common pattern of poor care coordination [5]. Furthermore, a more recent analysis by Penm et al. [47] found that poor primary care coordination was more likely to occur among patients younger than 65 years. Therefore findings from this study may be applicable to care coordination for age groups beyond older adults.

The study does possess several challenges of note. First, maintaining adequate statistical power will require that a sufficient number of patients experience acute care events outside the VHA. This is not desired, but necessary to observe natural care coordination processes in addition to those influenced by the event notifications. If there are fewer than expected non-VHA encounters, enrollment will need to be increased or the study timeframe will need to be lengthened. This is one reason why we added a second phase to incorporate individuals who are unable to be approached and recruited formally into the study. Such a design is atypical for trials. However, it is impossible to predict a priori which patients will have an acute encounter outside of the integrated VHA health system.

Furthermore, although we considered using a third, “usual care” arm for enrolled patients like most trials instead of the second phase, we ultimately deemed it potentially unethical to deny patients a potentially valuable event notification intervention. Ethical dilemmas with respect to randomization are common in biomedical informatics intervention studies [48], which often require quasi-experimental approaches [49]. Moreover, the number of primary care medical homes distributed across the two sites would limit statistical power if they were divided into thirds for randomization. Therefore we opted to retrospectively use patients who did not enroll in the study or who enrolled but had a non-system encounter outside the prospective monitoring period as a control arm. Doing so enables us to retain the robustness of randomization in arms 1 and 2 while maintaining statistical power with arm 3.

Another challenge for the study will be the implications of the findings. Should the care transitions intervention in combination with event notification prove to be superior, how can the VHA feasibly disseminate this approach? Furthermore, how can health systems economically sustain such an approach? The care transitions intervention requires active monitoring by a social worker or other care coordinator following discharge from the “out-of-network” facility. A home visit and phone monitoring may not be possible in every care transition. A challenge for our team will be to determine under what circumstances a superior but more intense intervention would be cost effective for the VHA or another health system.

## Data Availability

Data collection forms, including questionnaires as well as chart review templates, are available for inspection as well as use by other investigators. To receive a copy of these materials, please contact the corresponding author. Data collected for this trial from medical records as well as directly from Veterans is regulated by the VHA. Final datasets from this trial may be available to other researchers through a data use agreement (DUA) with non-federal entities. To inquire about data reuse, please contact the corresponding author.

## Abbreviations

ED: Emergency department
EHR: Electronic health record
HIE: Health information exchange
IHIE: Indiana Health Information Exchange
INPC: Indiana Network for Patient Care
RHIO: Regional Health Information Organization
VA: Veterans Administration
VAMC: Veterans Administration Medical Center
VHA: Veterans Health Administration
